# The Pittman Scholar Program for junior faculty recognition at the University of Alabama at Birmingham Heersink School of Medicine

**DOI:** 10.1080/10872981.2023.2182188

**Published:** 2023-03-01

**Authors:** Cayla Hurst, Toni R. Leeth, Etty N. Benveniste, Robert P. Kimberly, Craig Hoesley, LaKisha Mack, Mona N. Fouad, David A. Rogers, Selwyn M. Vickers, Anupam Agarwal

**Affiliations:** a Administrative Fellow, UAB Academic Medicine and Ambulatory Operations, Birmingham, AL, USA; b Strategic Planning and Administration for the Heersink School of Medicine, the University of Alabama at Birmingham, Birmingham, AL, USA; c Research for the Heersink School of Medicine, the University of Alabama at Birmingham, Birmingham, AL, USA; d Clinical and Translational Research for the Heersink School of Medicine, the University of Alabama at Birmingham, Birmingham, AL, USA; e Medical Education for the Heersink School of Medicine, the University of Alabama at Birmingham, Birmingham, AL, USA; f Administration and Finance for the Heersink School of Medicine, the University of Alabama at Birmingham, Birmingham, AL, USA; g Diversity and Inclusion for the Heersink School of Medicine, the University of Alabama at Birmingham, Birmingham, AL, USA; h Wellness Officer for UAB Medicine and the Heersink School of Medicine, the University of Alabama at Birmingham, Birmingham, AL, USA; i Medicine and Dean of the Heersink School of Medicine and Chief Executive Officer of the UAB Health System, the University of Alabama at Birmingham, Birmingham, AL, USA; j The Heersink School of Medicine, the University of Alabama at Birmingham, Birmingham, AL, USA

**Keywords:** Junior faculty recognition, scholar, faculty development, faculty productivity

## Abstract

The University of Alabama at Birmingham Heersink School of Medicine established the Pittman Scholars Program in 2015 to elevate scientific impact and to support the recruitment and retention of highly competitive junior faculty. The authors examined the impact of this program on research productivity and on faculty retention. The authors evaluated publications and extramural grant awards and available demographic data for the Pittman Scholars compared to all junior faculty in the Heersink School of Medicine. From 2015 to 2021, the program awarded a diverse group of 41 junior faculty members across the institution. For this cohort, ninety-four new extramural grants were awarded and 146 grant applications were submitted since the inception of the scholar award. Pittman Scholars published a total of 411 papers during the term of the award. The faculty retention rate of the scholars was 95%, comparable to that of all Heersink junior faculty, with 2 recipients being recruited to other institutions. The implementation of the Pittman Scholars Program has been an effective strategy to celebrate scientific impact and acknowledge junior faculty members as outstanding scientists at our institution. The Pittman Scholars award allows junior faculty to use funds for their research program, publications, collaborations, and career advancement. The Pittman Scholars are recognized at local, regional, and national levels for the work they are contributing to academic medicine. The program has served as an important pipeline faculty development program and an avenue for individual recognition for research-intensive faculty.

## Introduction

Recruiting and retaining talented early-career faculty members is a major emphasis for academic medical centers (AMCs). The challenges of a leaky pipeline in academic medicine, particularly for physician-scientists, continue to be significant and have been highlighted in several publications [1–4]. Diversifying the faculty workforce is also a priority for AMCs to ensure they reflect their community [5]. Faculty at the Associate Professor or Professor level have multiple opportunities for recognition at local, regional, and national levels, and most endowed positions at institutions are predominantly awarded to faculty in senior ranks.

In 2013 the University of Alabama at Birmingham (UAB) Heersink School of Medicine began a significant effort of transformation to address a decline in extramural funding, where it had fallen to 31^st^ in National Institutes of Health (NIH) funding, its lowest in over a decade [6]. This transformation included creating a secured and sustained Academic Enrichment Fund providing critical resources for reinvestment in the school, particularly in strategic priorities to boost research. The Pittman Scholars Program was a part of this comprehensive plan and was designed to support the scientific achievement of junior faculty.

### Description of the Pittman Scholars Program

The Pittman Scholars Program was established in 2015 to support the recruitment and retention of highly competitive junior faculty members in the UAB Heersink School of Medicine. The Pittman Scholars Program is named after the late James A. Pittman, MD. Dr. Pittman served as dean of the Heersink School of Medicine for 19 years (1973 to 1992) and was recognized for his ability to recruit and retain nationally and internationally known scientists and physicians to UAB. Each year, an application request is released and advertised through the Dean’s newsletter. The scholars are nominated by department chairs and receive funding of the equivalent of spendable income ($12,500/year) generated from a $250,000 endowed fund each year for three years and are designated as a Pittman Scholar to support their research or scholarly pursuits in basic or clinical sciences. The nomination package includes a cover letter (limited to one page) from the department chair along with the nominee’s resume and a one-page personal statement. Candidates are recognized as Pittman Scholars for the duration of their award (3 years). As a scholar, their primary responsibility is research, although teaching and some clinical duties are included. A junior faculty member can be nominated if they are a full-time Assistant Professor in the Heersink School of Medicine within the first five years of their initial appointment at this rank. Faculty members with highly competitive research programs judged by extramural funding and publications are eligible (Table 1).

**Table 1.** Eligibility criteria for the Pittman scholar award.

Eligibility Criteria
•Full-time assistant professors in the Heersink School of Medicine •Within the first 5 years of their initial appointment at the rank of assistant professor •Any prior appointment as an instructor does not count against the five-year limit •Primary responsibility is research, although teaching and some clinical duties are permissible •Faculty members with highly competitive research programs judged by extramural funding, high impact publications, and regional/national awards

Once all nominations are received, a panel of reviewers, which include former Pittman Scholars, review the applications to identify Pittman Scholars. Each application is reviewed by at least 6 reviewers and scored on a scale of 1–5 (1 being the best) based on the merits of the nominee particularly focused on high impact publications, extramural funding, and recognition with major awards at the regional/national level. Reviewers do not review applications from their respective departments to avoid any conflicts. Historically, 5 to 10 Pittman Scholars have been recognized each year. Funding may be used to support the research-related activities or scholarly enrichment of the faculty member. Since the Pittman Scholars Program inception in 2015, the program has supported 41 junior faculty at the Assistant Professor rank.

## Methods

Each Pittman Scholar submitted an annual progress report that included a list of publications, extramural support and grant applications. These documents also included a narrative section where the scholar describe how the award had allowed them to gain or maintain NIH or extramural funding, enhance collaborative interactions, produce high-impact publications, and along with a description of any unanticipated challenges. Details on how the funds have been used by category and description of the expenses in each category noting if the nature of expenses was research related or scholarly enrichment. Quantative analysis involved developing a count of totals where appropriate and the narratives were analyzed with attention to those that made an explicit linkage to the funding and subsequent scholarly achievement. To understand the Pittman Scholars Program’s impact on retention, retention rates of scholars were compared to that of all assistant professors in the Heersink School of Medicine.

## Results

### Demographic and quantitative findings

From the inception of this program in 2015, over 100 applications were received and 41

**Figure 1.**
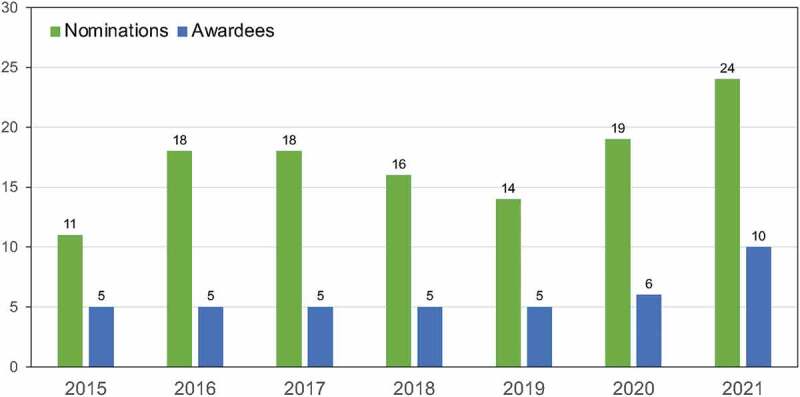
The number of nominations received and awards each year for the Pittman Scholar Program in the Heersink School of Medicine is shown.

Pittman Scholars have been supported (Figure 1), with a trending increase in applications per year during this time. For 2021, 10 scholars were awarded the Pittman Scholar designation. The Pittman Scholars are a diverse group of individuals and represent junior faculty across our institution. Over the past 6 years, both men and women of different races were represented (Table 2). The gender and ethnic diversity of the Pittman Scholars was similar to that of all Heersink School of Medicine junior faculty during the same time period, who were 54% male and 35% non-white. The scholar’s degrees vary from M.D., Ph.D., M.D./Ph.D., and Master of Science in Public Health (Table 2). The scholars have primary appointments in 15 departments (Table 3) and within the Department of Medicine in multiple divisions (cardiology, 10%; endocrinology, 10%; pulmonary, 7%; infectious diseases, 5%, nephrology, 5%; rheumatology, 2%). All applications were reviewed by reviewers from multiple departments (Table 4).

**Table 2.** Demographics of Pittman Scholars.

Scholars by Gender	
Male	59%
Female	41%
***Scholars by Race***	
Asian	22%
Black or African American	5%
Hispanic or Latino	5%
Two or More Races	2%
White	66%
***Scholars by Discipline***	
PhD	68%
MD	22%
MD PhD	7%
MD MSPH	3%

**Table 3.** Departmental affiliation of scholars.

Biochemistry and Molecular Genetics	2%
Cell, Developmental & Integrative Biology	7%
Genetics	2%
Medicine	39%
Microbiology	2%
Neurobiology	5%
Neurology	10%
Obstetrics & Gynecology	2%
Pathology	7%
Pediatrics	5%
Physical Medicine & Rehabilitation	2%
Psychiatry	2%
Radiology	2%
Surgery	7%
Urology	2%

**Table 4.** Departmental affiliation of reviewers.

Cell, Developmental and Integrative Biology	13%
Genetics	4%
Medicine	33%
Neurobiology	4%
Neurology	17%
Obstetrics & Gynecology	4%
Pathology	8%
Physical Medicine & Rehabilitation	4%
Psychiatry	4%
Surgery	4%
Urology	4%

From 2015 to 2021, Pittman Scholars had a total of 106 extramural grants active at the time of receipt of the scholar award. Ninety-four new extramural grants were awarded since the inception of the scholar award, and 146 extramural grant applications were submitted since the inception of the scholar award. Overall, a total of 411 papers were published after receiving the Pittman Scholar award (Table 5).

**Table 5.** Pittman scholar report achievements from 2015 to 2021.

Achievement:	Total:
Extramural Support active at the time of receipt of the scholar award	106
New extramural support that has been awarded since the inception of the scholar award	94
Extramural support applications submitted since the inception of the scholar award	146
Number of Publications since Scholar Award	411

### Impact on the scholars

The Pittman Scholars Program has impacted early-career faculty and has provided a positive return on investment with faculty retention, extramural funding, and publications from the recipients. Recipients have worked on a multitude of collaborative interactions that have enhanced extramural funding and publications. Scholars reported that these collaborations resulted in, but were not limited to, NIH R01 and R21 awards, K awards, and career development awards from the Department of Veterans Affairs and other funding agencies. The award has provided scholars a platform for a support system and recognition at an institutional level to establish interdisciplinary collaborations. As a group of scholars, they have enhanced networking opportunities.

A few select comments from recipients are listed below:

*“The Pittman scholar award supported research funded by an NIH K23 project, lead to a collaborative R01, a D43 submission, publication of 6 manuscripts per year and attendance to scientific meetings to present study findings.”* Another scholar noted, *“The award has assisted in maintaining laboratory funding and has been able to contribute to continued training of graduate students and post-doctoral fellows in advancing research projects”*. Another recipient stated, *“The Pittman Scholar Award has been used to allow their laboratory to harness whole-genome sequencing approaches to generate novel pilot data. The award also contributed to the ability to attend conferences to allow for further networking, chances for funding, recruitment to laboratories, and opportunities for external collaborations.”* Yet another scholar mentioned, *“The Pittman Scholar award has greatly assisted me in advancing my research on surgical disparities and health literacy. I have been able to fund assistants to help measure health literacy in the clinic and to establish a health literacy database, this work lead to a K12 award, and is now supported by a K23 grant that was funded on its first submission.” “Pittman Scholars award allows for flexibility for new techniques, executing pilot experiments, and ultimately discovering new areas of exploration for future grants and publications.”*

### Impact on the institution

The faculty retention rate was 95%, with only 2 of the 41 scholars being recruited elsewhere since the initiation of this program. This is the same as the faculty retention rate of all Heersink School of Medicine junior faculty for the same time period (95%). Both faculty members who left the institution continue to pursue scholarly research at other academic institutions. Pittman Scholars are invited to participate in the annual research retreat for the Heersink School of Medicine where they present their research work in the form of posters. Although a relatively a new program, several recipients have been recognized by membership and awards in prestigious societies such as the American Society for Clinical Investigation and other specialty-specific organizations.

## Discussion

The results of this work highlight the impact and success of the Pittman Scholars Program for early-career faculty at the Heersink School of Medicine over the past several years. The program is intended to provide an opportunity for junior faculty members to advance their careers. At the institutional level, the goals of the program were to elevate scientific impact and recruit and retain talented junior faculty. The Pittman Scholars Program has reached these goals by the reputation of the program and the productivity of the recipients, faculty recognition, and faculty retention.

The Pittman Scholars have shown excellent productivity through extramural funding and publications while in the program over the past six years. With over 400 publications, scholars serve in a variety of roles including principal investigators, mentors, co-investigators, collaborators, and more. In addition to the productivity of the Pittman Scholars, this program was able to recognize junior faculty across 15 departments at our institution. This recognition was aided with the recruitment and retention of junior faculty within the institution, for which Dr. James A. Pittman strived.

Paller and Cerra described a seed grants program (1 year, one-time award of $25,000) to investigators to initiate a new area of research or for innovative projects over a 6-year period at the University of Minnesota [7]. This program was open to all faculty and not restricted to early career faculty alone. The reported financial yield for this seed program investment was 560%. A recent study described an intramural grants program for early career faculty members providing research support for junior faculty members resulting in a positive influence on career trajectory [8]. This study reported on a comparison of career development and retention through a mentoring program. Though different in terms of the approach, the results suggested that even small amounts of research support received early in a career can benefit the faculty and university as a whole, and are consistent with our Pittman Scholars Program.

### Limitations

Given that the Pittman Scholars Program is a relatively new and single-institution program with a small sample size, the recipients have embraced the opportunity and the award has truly impacted each recipient and the institution. We focused on scholarly research productivity and did not include teaching or clinical service-related accomplishments and hope to add these metrics going forward.

In conclusion, the implementation of the Pittman Scholars Program has been an effective strategy to recognize junior faculty members at our institution. The award allows junior faculty members to use flexible funds for research support, publications, collaborations, and career advancement. The Pittman Scholars Program provides multiple opportunities for junior faculty members to be recognized at local, regional, and national levels, which are typically only for senior rank faculty. The success of the Pittman Scholars Program highlights the value of allocating resources to junior faculty for their careers as well as their growth within the institution.
